# Inadequate oral anticoagulation with warfarin in women with cerebrovascular event and history of atrial fibrillation: the FibStroke study

**DOI:** 10.1080/07853890.2021.1875499

**Published:** 2021-01-21

**Authors:** Aissa Bah, Ilpo Nuotio, Antti Palomäki, Pirjo Mustonen, Tuomas Kiviniemi, Antti Ylitalo, Päivi Hartikainen, K. E. Juhani Airaksinen, Juha E. K. Hartikainen

**Affiliations:** a Heart Center, Kuopio University Hospital and University of Eastern Finland, Kuopio, Finland; b Department of Acute Internal Medicine, Turku University Hospital, Turku, Finland; c Heart Center, Turku University Hospital and University of Turku, Turku, Finland; d Department of Medicine, Keski-Suomi Central Hospital, Jyväskylä, Finland; e Faculty of Information Technology, Jyväskylä University, Jyväskylä, Finland; f Heart Center, Satakunta Central Hospital, Pori, Finland; g Neurocenter, Neurology, Kuopio University Hospital, Kuopio, Finland

**Keywords:** Atrial fibrillation (AF), oral anticoagulation (OAC), CHADS_2_, CHA_2_DS_2_-VASc, sex

## Abstract

**Background:**

Women with atrial fibrillation (AF) may be treated less actively with oral anticoagulation (OAC) than men.

**Patients and methods:**

We assessed sex differences in the implementation of stroke risk stratification with CHADS_2_ and CHA_2_DS_2_-VASc scores and reasons not to use OAC in 1747 AF patients suffering their first cerebrovascular event after the AF diagnosis.

**Results:**

Women were older and had more often a high stroke risk (CHADS_2_/CHA_2_DS_2_-VASc ≥2) than men (*p* < .001). On admission, 46.4% of women and 48.2% of men were on OAC with no sex difference (*p* = .437). However, of patients without OAC, 74.4% of women and 49.5% of men should have been on OAC based on CHADS_2_/CHA_2_DS_2_-VASc ≥2 (*p* < .001). Conversely, 34.8% of men and 17.5% of women on OAC had a low or moderate risk (CHADS_2_/CHA_2_DS_2_-VASc 0–1, *p* < .001). A valid reason to omit OAC was reported in 38.6% of patients and less often in women (*p* < .001).

**Conclusions:**

OAC was underused in high-risk AF patients, particularly women, but prescribed often in men with low or moderate stroke risk. Reasons for omitting OAC treatment were poorly reported, particularly for women.

**KEY MESSAGE**
- Women were at higher stroke risk, but were less often treated with oral anticoagulation (OAC).
- Men were more often on OAC at low or moderate stroke risk.
- Reasons for omitting guideline based OAC were poorly reported, particularly for women.

## Introduction

Atrial fibrillation (AF) is the most common sustained arrhythmia and its prevalence increases with age [1,2]. A 2.5-fold increase in AF prevalence has been estimated in the United States by 2050 [3] as well as an 18% global rise in disability-adjusted life-years [4]. AF is more common in men in general, but in the elderly the proportion of women increases due to their longer survival [5] and women have more comorbidities and a higher thromboembolic risk than men [1,2].

Oral anticoagulation (OAC) therapy reduces the risk of thromboembolic complications by two-thirds [6]. In addition, strokes that occur during proper anticoagulation are not as severe as those without adequate therapy [7]. Guidelines recommend OAC for AF patients with risk factors for stroke unless contraindicated [1,2]. Despite solid evidence, guidelines and effective treatment available, there is substantial heterogeneity and inappropriateness in the use of OAC worldwide [8]. OAC is often underused in community practice [8,9] and discontinuation of OAC after the first years is a major problem [10]. Particularly, there are reports suggesting that women with AF are treated less actively than men both with anticoagulation [11,12] as well as with rhythm control therapy [13].

This study aims to investigate whether there are gender differences in the implementation of risk stratification and the use of OAC in patients with a history of AF and subsequently suffering a cerebrovascular event (stroke or intracranial haemorrhage). Particularly, we evaluated the time-period between the diagnosis of AF and the cerebrovascular event. We also assessed the reasons for omitting OAC in AF patients with a high thromboembolic risk.

## Materials and methods

The FibStroke study is a multicentre study, which is part of an ongoing study program assessing cerebrovascular thrombotic and bleeding complications related to AF in Finland (ClinicalTrials.gov Identifier: NCT02146040) [9,14,15].

### Study population

The study population consists of all patients admitted to two university hospitals and two central hospitals from 2003 through 2012 with a diagnosis of AF (ICD-10 code I48) and stroke, transient ischaemic attack or intracranial haemorrhage [16]. The appropriate ICD-10 codes were identified from the hospital discharge registries. This prespecified substudy included 1747 patients (1) ≥18 years of age with (2) previously known history of AF (paroxysmal, persistent or permanent) and either (3) intracranial haemorrhage or (4) first-ever ischaemic stroke or TIA occurring after the diagnosis of AF. Each hospital is the only referral hospital responsible for the acute care of patients with cardiac and neurologic events in their catchment area and thus, ensures that the patient had an established diagnosis of AF and that the index event was indeed the patient’s first cerebrovascular event.

Patients’ clinical characteristics, date of AF diagnosis, medical history and laboratory values during admission as well as medication prior to and at the time of admission were collected by reviewing the individual secondary care medical records. Reasons for not being on OAC were identified from patient records and divided into (1) valid reasons (CHADS_2_ or CHA_2_DS_2_-VASc score 0–1, prior intracerebral haemorrhage), (2) relative reasons (dementia, prior gastrointestinal bleed, excessive alcohol consumption and history of frequent falls), (3) non-valid reasons (anaemia, patient refusal, small stroke risk, paroxysmal AF and restored sinus rhythm after electrical cardioversion) or (4) undocumented reasons.

At the time of our study, CHADS_2_ score was used to assess thromboembolic risk until 31 December 2009 and CHA_2_DS_2_-VASc score from 1 January 2010 onwards [1,2]. The index cerebrovascular event was not included in the calculation of CHADS_2_/CHA_2_DS_2_-VASc scores. A high stroke risk score was defined as CHADS_2_ or CHA_2_DS_2_-VASc score ≥2. INR data were available only from the last 30 days prior to the cerebrovascular event. Thus, a modified HAS-BLED score omitting labile INR was used to assess bleeding risk. Direct oral anticoagulants (DOACs) were used in less than 0.5% of patients. Thus, they were excluded from the analyses.

### Stroke and intracranial haemorrhage

All patients underwent computed tomography or magnetic resonance imaging during the index hospitalization. Thrombotic events were defined as (1) a stroke documented clinically and considered definite by a neurologist and confirmed by imaging (computed tomography or magnetic resonance imaging) or (2) a transient ischaemic attack defined according to Albers et al. [17] and diagnosed clinically by a neurologist. Intracranial haemorrhage events including intracerebral haemorrhage, subdural haematoma and subarachnoid bleeding were diagnosed by the neurologist and confirmed by imaging.

### Statistical analysis

Comparisons between groups were performed with the Chi-square or Fisher’s exact test for categorical variables and Student’s *t*-test and Mann–Whitney’s *U*-test for analysis of continuous data as appropriate. Time-specific calculations were made with the Mann–Whitney *U*-test and reported as the median and interquartile ranges. In addition, we evaluated the use of OAC in women and men by calculating odds ratios (ORs) between women and men in respect with (1) the use of OAC in patients with high risk score as well as (2) the prevalence of patients with high risk score among those on and not on OAC. Two-sided differences at *p* < .05 were considered statistically significant. Statistical analyses were performed using version Statistics 22 of IBM SPSS (IBM Corporation and Others 1989, 2013, Armonk, NY).

### Institutional review board

This study conforms to the Declaration of Helsinki as revised in 2013 and the protocol was approved by the Ethics Committees of the Hospital District of Southwest Finland and the National Institute for Health and Welfare. Informed consent was not required because of the register-based nature of the study and all patient data were anonymized.

## Results

### Clinical characteristics of patients

Women were approximately seven years older than men and approximately three quarters of women and half of men were at least 75 years old (Table 1). Women had more comorbidities such as hypertension, congestive heart failure, a cardiac pacemaker and renal dysfunction. Men had more frequently a history of myocardial infarction, alcohol overuse and liver disease than women. The index cerebrovascular event was stroke more often for women whereas intracranial bleeds were more frequent among men.

**Table 1.** Clinical characteristics of the patient population at the time of cerebrovascular event.

	Women	Men	All	*p* Value
	(*n* = 960)	(*n* = 787)	(*n* = 1747)	
Age	79.8 ± 8.5	73.5 ± 10.6	77.0 ± 10.0	<.001
Age 65–75 years	190 (19.8)	240 (30.5)	430 (24.6)	<.001
Age ≥ 75 years	716 (74.6)	381 (48.3)	1096 (62.7)	<.001
Hypertension	674 (70.3)	492 (62.5)	1166 (66.8)	.001
Heart failure	220 (22.9)	136 (17.3)	356 (20.4)	.004
Severe renal impairment*	43 (4.6)	18 (2.3)	61 (3.5)	.012
Anaemia (haemoglobin <10 g/dL)	34 (3.6)	14 (1.8)	48 (2.8)	.024
Chronic liver disease	2 (0.2)	16 (2.0)	18 (1.0)	<.001
Alcohol overuse	17 (1.8)	103 (13.1)	120 (6.9)	<.001
Prior myocardial infarction	134 (14.0)	163 (20.7)	297 (17.0)	<.001
Prior bleeding	70 (7.3)	47 (6.3)	117 (6.7)	.270
Permanent pacemaker	95 (9.9)	55 (7.0)	150 (8.6)	.032
Biological valve prosthesis	11 (1.1)	11 (1.4)	22 (1.5)	.068
Paroxysmal AF	448 (46.7)	324 (41.2)	772 (44.2)	.021
Permanent or persistent AF	411 (42.8)	359 (45.6)	770 (44.1)	.240
Stroke	653 (68.0)	461 (58.6)	1114 (63.8)	<.001
TIA	162 (16.9)	160 (20.4)	322 (18.4)	.062
Intracranial haemorrhage	147 (15.3)	169 (21.5)	316 (18.1)	.004
Warfarin	445 (46.4)	379 (48.2)	824 (47.2)	.437
Aspirin	328 (34.4)	295 (37.5)	623 (35.8)	.173
INR (admission)	2.0 ± 1.1	2.1 ± 1.1	2.1 ± 1.1	.146
	1.9 [1.3–2.5]	2.0 [1.4–2.6]	1.9 [1.3–2.6]	
INR 2–3 (of those on OAC)	206 (45.0)	189 (48.6)	395 (46.6)	.446
CHADS_2_ (until end 2009)	1.8 ± 1.0	1.4 ± 1.0	1.6 ± 1.0	<.001
CHADS_2_ ≥2	364 (64.7)	198 (43.1)	562 (55.0)	<.001
CHA_2_DS_2_-VASc (from 2010)	4.2 ± 1.3	2.7 ± 1.4	3.5 ± 1.5	<.001
CHA_2_DS_2_-VASc ≥2	390 (98.2)	258 (78.7)	648 (89.4)	<.001
CHADS_2_/CHA_2_DS_2_-VASc ≥2	754 (78.5)	456 (57.9)	1210 (69.3)	<.001
HAS-BLED*	2.3 ± 0.9	2.1 ± 1.0	2.2 ± 0.9	.001

AF: atrial fibrillation; TIA: transient ischaemic attack; CHADS_2_: congestive heart failure, hypertension, age ≥75 years, diabetes, prior stroke; CHA_2_DS_2_-VASc: congestive heart failure, hypertension, age ≥75 years (two points), diabetes, prior stroke/transient ischaemic attack/systemic embolism (two points), associated Vascular disease, age 65–74 years and female sex category; HAS-BLED* (labile INR omitted): hypertension, abnormal liver or kidney function, prior stroke, bleeding history or predisposition, labile INR (omitted), elderly and concomitant drugs; severe renal dysfunction*: estimated glomerular filtration rate (Chronic Kidney Disease Epidemiology Collaboration)<30 ml/min/1.73 m^2^.

The values denote mean (standard deviation), median [interquartile range] or *n* (%). *p* Value refers to women vs. men.

### Risk stratification

At the time of the index cerebrovascular event, both CHADS_2_ score (until end 2009) and CHA_2_DS_2_-VASc score (from 2010 onwards) were higher in women (Table 1). Correspondingly, a high thromboembolic risk (CHADS_2_/CHA_2_DS_2_-VASc score ≥2) was found more often in women (78.5%) compared to men (57.9%). CHADS_2_ score ≥2 was present in 64.7% of women and in 43.1% of men. The difference was even more pronounced for the CHA_2_DS_2_-VASc score: CHA_2_DS_2_-VASc score ≥2 was present practically in all (98.2%) women in comparison with 78.7% of men. Women had also slightly higher HAS-BLED scores (Table 1).

### Oral anticoagulation

At the time of the cerebrovascular event approximately half of the patients were on OAC therapy (warfarin) with no sex difference (Table 1). Nor was there any significant sex-related difference in INR levels during admission: about half of the patients had INR within the therapeutic range (2.0–3.0) (Table 1).

**Figure 1.**
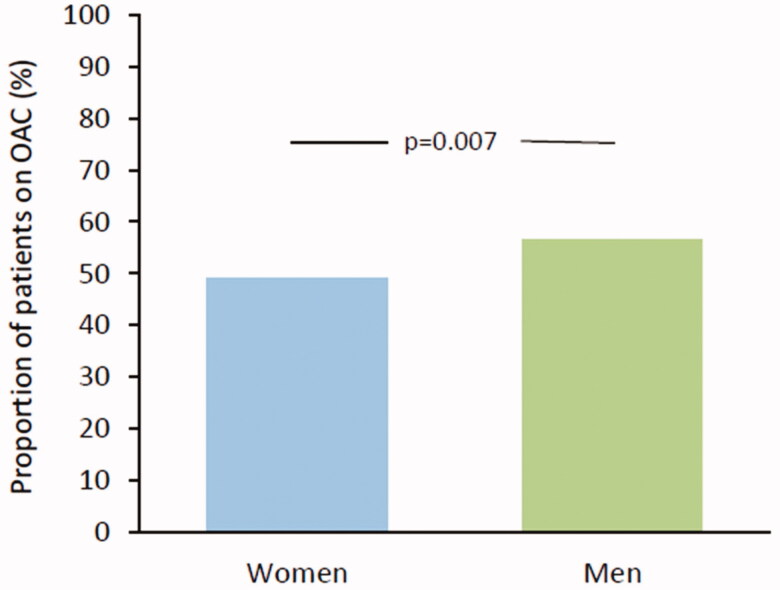
Oral anticoagulation in patients with high stroke risk (CHADS_2_/CHA_2_DS_2_-VASc ≥2).

However, when risk stratification was taken into account, women with a high-risk score (CHADS_2_/CHA_2_DS_2_-VASc ≥2) were significantly less often on OAC than men: 49.2% of women and 56.7% of men were on OAC (OR 0.80, 95% CI 0.50–0.93, *p* = .011) (Figure 1). During the CHADS_2_ era, 44.8% of women and 48.0% of men with a high stroke risk were on OAC with no difference between the sexes (OR 0.88, 95% CI 0.62–1.25, *p* = .467). However, during the CHA_2_DS_2_-VASc era 53.3% of women with high risk were on OAC compared with 63.4% of men (OR 0.66, 95% CI 0.48–0.91, *p* = .011).

**Figure 2.**
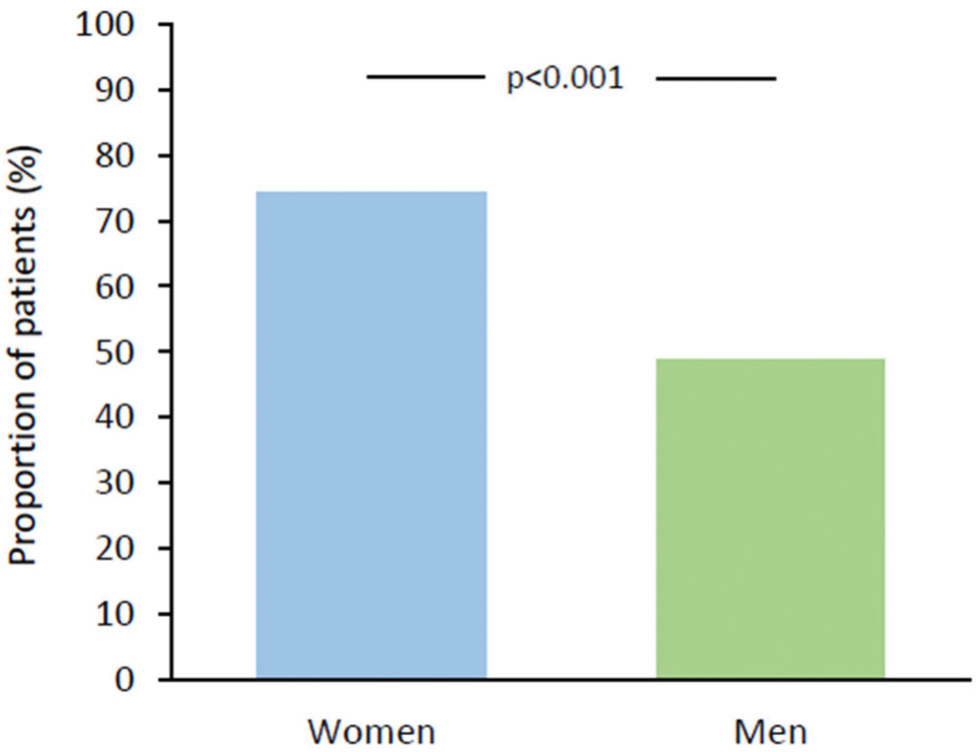
Proportion of high-risk patients (CHADS_2_/CHA_2_DS_2_-VASc ≥2) among those not on oral anticoagulation.

In addition, among patients without OAC a high-risk score was present more often in women (74.4%) than in men (49.5) (*p* < .001) (Figure 2). During the CHADS_2_ era, 61.5% of women and 38.1% of men not on OAC had a high risk score (*p* < .001) and during the CHA_2_DS_2_-VASc era a high risk score was present in 96.8% of women and 66.2% of men (*p* < .001).

**Figure 3.**
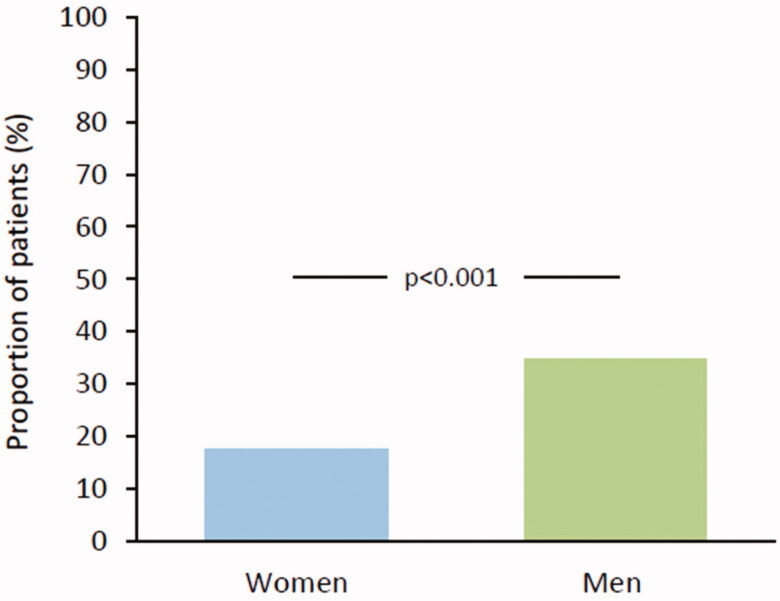
Proportion of low and moderate risk patients (CHADS_2_/CHA_2_DS_2_-VASc 0–1) among those on oral anticoagulation.

The use of OAC treatment was inconsistent in patients with a low or moderate stroke risk as well (CHADS_2_/CHA_2_DS_2_-VASc score 0–1): A total of 34.8% of men on OAC had low or moderate risk compared to 17.5% of women (*p* < .001) (Figure 3).

### Reasons for not being anticoagulated

A valid reason for omitting OAC was recorded in 38.6% of patients with a marked difference between sexes: approximately one quarter of women had a valid reason not to be prescribed OAC compared with half of men (Table 2). If only CHADS_2_/CHA_2_DS_2_-VASc 0 and intracranial haemorrhage were accepted as valid reasons to withhold OAC, 8.9% of women and 19.6% of men presented with a valid reason.

**Table 2.** Reasons for not being anticoagulated in patients with AF diagnosed before cerebrovascular event.

	Women	Men	All	*p* Value
	(*n* = 515)	(*n* = 408)	(*n* = 923)	
Valid reason	139 (27.0)	217 (53.2)	356 (38.6)	<.001
CHADS_2_/CHA_2_DS_2_-VASc 0–1	132 (25.6)	210 (51.5)	342 (37.1)	<.001
CHADS_2_ 0	33 (6.4)	57 (14.0)	90 (9.8)	<.001
CHADS_2_ 1	93 (18.1)	104 (25.5)	197 (21.3)	.005
CHA_2_DS_2_-VASc 0	6 (1.2)	16 (3.9)	22 (2.4)	.004
CHA_2_DS_2_-VASc 1	0 (0.0)	33 (8.1)	33 (3.6)	<.001
Intracranial haemorrhage	7 (1.4)	7 (1.7)	14 (1.5)	.657
Relative reason	35 (6.8)	37 (9.1)	72 (7.8)	.197
Non-valid reason	82 (15.9)	57 (14.0)	139 (15.1)	.755
Undocumented reason	259 (50.3)	97 (23.8)	356 (38.6)	.898

Valid reason: CHADS_2_/CHA_2_DS_2_-VASc < 2 or intracranial haemorrhage; relative reason: dementia, prior gastrointestinal bleed, excess alcohol intake, frequent falls; non-valid reason: anaemia, patient refusal, small stroke risk, paroxysmal AF and restoration of sinus rhythm after electrical cardioversion.

CHADS_2_ and CHA_2_DS_2_-VASc, see Table 1. The values denote *n* (%). *p* Value refers to women vs. men.

When evaluating patients with high stroke risk (CHADS_2_/CHA_2_DS_2_-VASc score ≥2) and not on OAC, women not on OAC were older than men (*p* = .008), had more often a high HAS-BLED score (*p* = .041) and were more often on aspirin (*p* = .002) (Table 3). A majority of patients not on OAC had a history of paroxysmal AF and about half of them were >75 years old with no difference between the sexes.

**Table 3.** Clinical characteristic on patients with CHADS_2_ and CHA_2_DS_2_-VASc ≥ 2 and not on OAC.

	Women	Men	All	*p* Value
Age	82.2 ± 8.2	78.6 ± 8.4	81.0 ± 8.4	.008
Age ≥ 75 years	335 (51.5)	150 (45.7)	485 (49.5)	.091
HAS-BLED ≥3	234 (73.1)	132 (64.7)	366 (69.8)	.041
Paroxysmal AF	215 (68.3)	108 (65.1)	323 (67.2)	.478
Aspirin use	219 (86.2)	139 (74.7)	358 (81.4)	.002

AF: atrial fibrillation.

CHADS_2_ and CHA_2_DS_2_-VASc, see Table 1. The values denote mean ± SD (age) or *n* (%). *p* Value refers to women vs. men.

## Discussion

The main finding of our study was that stroke risk evaluation in AF patients was performed poorly resulting in underuse of OAC particularly in women. Three quarters of women and half of men were not using OAC at the time of the cerebrovascular event in spite of guideline-based indication (CHADS_2_/CHA_2_DS_2_-VASc ≥2) for OAC. Second, futile use of OAC was frequent in younger men with only low or moderate stroke risk. Reasons for omitting guideline based OAC were poorly reported, particularly for women.

The 2006 ESC guidelines were the first to recommend routine use of risk stratification scores to guide OAC initiation. At the time of our study, guidelines recommended OAC for AF patients with CHADS_2_ ≥2 (until 2009) or CHA_2_DS_2_-VASc ≥2 (after 2010) for women and men [1,2]. In the current ESC 2020 guidelines, all women are given one risk point and OAC is recommended with CHA_2_DS_2_-VASc ≥3 for women and ≥2 for men. This was not applied in our study and the adherence to risk stratification was based on the ESC 2006 and 2010 guidelines, i.e. those valid at the time of the cerebrovascular event. In line with earlier reports on real-life use of OAC, the implementation of these recommendations was inadequate also in AF patients suffering a cerebrovascular event [8,11,18,19]. Also in accordance with earlier reports, only half of the patients in our study had an INR within the therapeutic target [8,19].

Our study shows that there were sex-related differences in the guideline-based use of OAC. Almost three quarters of women not using OAC at the time of the index cerebrovascular event had a high-risk score for stroke. The sex difference in OAC use became even more marked after 2010 when the CHA_2_DS_2_-VASc score was implemented and women aged 65–75 years are reclassified from low to high risk category (from 0 to 2) [2]. It seems that this change did not penetrate clinical practice [7,19,20]. During the CHA_2_DS_2_-VASc era 98% of women belonged to the high stroke risk category (score ≥2) but only 53% were on OAC.

The effect of age on the risk evaluation is more pronounced in the CHA_2_DS_2_-VASc score. In the CHADS_2_ era, age ≥75 years merited one risk point, whereas in the CHA_2_DS_2_-VASc era, age 65–74 years scores one point and age ≥75 years two points. In our study, women were older than men increasing the stroke risk in women, but this was not reflected in the more frequent use of OAC in older women. Earlier studies have reported that not only OAC but also rhythm control strategy are less often used in women than in men with AF [13,21,22].

Valid reasons for not prescribing OAC (intracranial haemorrhage and CHADS_2_/CHA_2_DS_2_-VASc 0–1) were identified in half of men but only in a quarter of women. The risk of stroke in patients with CHADS_2_/CHA_2_DS_2_-VASc 0 is very low and these patients do not need OAC. Patients with CHADS_2_/CHA_2_DS_2_-VASc score 1 are at moderate risk and the current ESC guidelines (2020) recommend considering OAC in these patients [23]. If intracranial haemorrhage and CHADS_2_/CHA_2_DS_2_-VASc 0 are used as justifiable reasons to omit OAC, only 14% of patients presented with such a valid reason to omit OAC. These findings are in line with Xian et al. who reported that the reason for not using OAC therapy was documented only in one-third of high-risk AF patients [19].

In the present study, the most common non-valid reason to omit OAC was paroxysmal AF with successful cardioversion to sinus rhythm. Two-thirds, both women and men, not on OAC had a history of paroxysmal AF. Paroxysmal AF is, however, associated with an increased stroke risk and the risk is considered to be similar to permanent or persistent AF [24]. One possible explanation for omitting OAC is older age and frailty [25]. In our study, OAC was deferred particularly in elderly women. Although old age increases the risk of bleeding, it is also a strong predictor of stroke [26]. Therefore, the benefits of stroke prevention usually overweigh the risk of bleeding also in older patients [27].

Stroke risk and bleeding risks often overlap and almost three quarters of women not being on OAC had HAS-BLED score ≥3 [28]. High bleeding risk should not automatically result in withholding OAC, but in the elimination of modifiable bleeding risk factors such as hypertension, non-steroidal anti-inflammatory drugs and alcohol use [23].

Potential reasons leading the patient to discontinue OAC are warfarin side effects, poor INR control and minor bleedings, which may not be recorded in the patient files. In the Re-LY trial, the incidence of minor bleedings in the warfarin group was 16.2% per year and 10.2% of patients discontinued warfarin therapy at 1-year follow-up [29].

Withholding OAC seems often to result in the prescription of aspirin. A majority of patients not on OAC were using aspirin in spite of the fact that the bleeding risk is similar to OAC particularly in the elderly with minimal effect on thromboembolic risk [30].

One important finding of our study was the frequent use of OAC in low and moderate risk patients, particularly in men, which is in line with previous reports [31,32]. One-third of men using OAC were at low or moderate risk (CHADS_2_ or CHA_2_DS_2_-VASc 0–1). Unfortunately, data regarding reasons for initiation of OAC in low and moderate risk patients were not collected in our study.

## Limitations

The retrospective nature is a limitation of the current study. The data were derived from hospital (secondary care) medical records. Thus, we do not have data from primary care for example on discontinuation of OAC and reasons leading to discontinuation. Prescription of OAC was always at the treating physician’s discretion and may have been affected by factors not written in the patient records. This, however, is one of the main results of the report and indicates the need for assessing valid reasons for initiation/withdrawing OAC in clinical practice. The strengths of the study include the identification of all consecutive stroke and TIA patients with a diagnosis of AF from reliable hospital discharge records and the thorough individual case by case review of patient records. We also included only patients living in the catchment area of the participating hospitals. Thus, medical history was well captured in our registry. INR data were collected only from the last 30 days prior to the cerebrovascular event.

Warfarin was the most commonly used OAC during the study period while DOACs are currently the dominant OAC therapy. However, the fundamental question, when to start OAC, remains also in the DOAC era. At present, there is no evidence to suggest that DOACs or new guidelines have changed the sex-gap between women and men in the treatment of AF. A recent EHRA position paper summarizes: “Sex-specific barriers to the implementation of contemporary AF guidelines and the use of guideline-recommended OAC therapy need to be identified and addressed” [21].

## Conclusions

Our results suggest that OAC is underused in high-risk AF patients, particularly women, and often prescribed in men with low or moderate stroke risk. In addition, the decision to omit OAC was rarely based on risk stratification scores and contemporary guidelines. Reasons for not being anticoagulated were poorly justified, particularly in women. These findings underline the need for improving the use of risk scores and OAC, especially in women with AF.

## Data Availability

Access to data is regulated by Finnish law. Data are available from the Turku University Hospital for researchers who meet the criteria as required by the Finnish law for access to confidential data. Contact person who will distribute data upon request to qualified researchers: Tuija Vasankari, Heart Centre, Turku University Hospital, PO BOX 52, FIN-20521 Turku, Finland; tuija.vasankari@tyks.fi.
